# Don't Shoot the Messenger

**DOI:** 10.1371/journal.pntd.0004166

**Published:** 2015-10-22

**Authors:** Judd L. Walson

**Affiliations:** Departments of Global Health, Medicine, Pediatrics and Epidemiology, University of Washington, Seattle, Washington, United States of America; University of Queensland, AUSTRALIA

*“It is better to debate a question without settling it than to settle a question without debating it*.*”*—*Joseph Joubert*

The incredible fervor surrounding the recent publication of articles calling into question the evidence of benefit for mass deworming treatment highlights the passion and commitment of the neglected tropical disease community. This is not simply an academic debate. This conversation highlights very real tensions within the global health community between the need for continued advocacy to ensure that individuals in some of the poorest and most marginalized communities continue to benefit from a proven interventional strategy and the need for policymakers and planners to be able to determine priorities for resource allocation. The very fact that this passionate debate is occurring is clear evidence of the need for more robust data to inform this conversation. In this editorial, I present my own personal reflections and thoughts regarding what I consider to be an important moment of opportunity for our community.

Decision making in public health is complex. Funding for global health appears to be stabilizing, and both policymakers and funders are being faced with difficult decisions regarding the prioritization of resources [1]. Increasingly, efforts are being made to ensure that available health funding is being allocated based on sound evidence-based assessments of impact. An improved understanding of the global burden of disease through efforts by groups such as the Child Health Epidemiology Reference Group (CHERG) and the Institute for Health Metrics and Evaluation (IHME), combined with economic analyses from groups such as Disease Control Priorities, Edition 3 (DCP3), seek to prioritize which health interventions are likely to have greatest impact by attempting to make the best use of available evidence [2–4]. However, even as some argue, as noted by Hicks, Kremer, and Miguel in this edition of *PLOS Neglected Tropical Diseases*, that “…the available evidence does suggest that mass deworming treatment should remain a policy priority in endemic regions,” we must also acknowledge that the available evidence does not uniformly suggest benefit and, at best, appears to demonstrate fairly modest impact only among those with documented infection [5,6].

Advocates of mass drug administration point out that such a delivery strategy is not predicated on the overall population benefit but is based on the logistical and practical benefits of treating everyone instead of attempting to diagnose and treat those with documented infection. However, treating all children in an effort to reach those with worms does cost governments and donors. Even with the massive donations of drugs to treat soil-transmitted helminths (STH), there are human and economic resources required to deliver this intervention at scale. It is critical that policymakers understand these costs and the gains that result from the intervention. Again, the lack of consistent and reliable data on costs from STH control programs precludes an accurate assessment of the relative cost-benefit of deworming as compared to alternative public health interventions [7]. As much as we need to strongly advocate for the continued need to treat children at risk of poor outcomes, we must also recognize that resource allocation must be based on comparative evidence assessment, and we must acknowledge that we lack robust evidence of the population benefits of STH control. We must continue to highlight the comments of de Silva et al., that “lack of evidence to support effectiveness cannot be considered as evidence of ineffectiveness” [8], but we also need to encourage policymakers to rely on strong evidence of benefit for planning and resource allocation and then work to ensure that we provide clear evidence of these benefits and their true costs.

Part of the challenge of documenting the benefits of deworming results from the types of outcomes we are attempting to measure. When evaluated, some objective endpoints, such as weight gain, do appear to be improved by deworming interventions in children who are documented to be infected [5]. However, as pointed out in many of the criticisms of the interpretation of this evidence, the inclusion of many children without helminth infection dilutes any potential effect size, making it difficult to observe consistent benefit. History has also placed the deworming community in a unique position. Deworming as a public health intervention began long before randomized trials were advocated as the single “gold standard” for evaluating the benefits of health interventions and well in advance of the establishment of the Cochrane Collaboration. As a result, much of the evidence for impact of deworming comes from observational studies. While this observational evidence suggests that deworming children with worms does result in significant health benefits, observational studies are subject to multiple potential sources of bias and may overestimate effect [9]. In addition, although some data from randomized trials of deworming are available, most of these trials have been limited by short follow-up times and have not included sufficient numbers of infected individuals to achieve adequate power to accurately assess outcomes of interest [10]. We should not be surprised by the conclusions of the Cochrane Collaboration. Instead, we should acknowledge the limited number of high-quality studies assessing the impact of deworming and the methodological flaws that result in the conclusions of the Cochrane review [5].

Many of the outcomes we seek to evaluate are also difficult to ascertain, including markers of early childhood development, such as school performance and cognitive function. These outcomes are also often undervalued in calculations of disease burden [11]. However, there is a global shift toward an improved appreciation of the need to better assess early childhood development as a critical component of disease burden and of interventional impact. As the global community moves toward the Sustainable Development Goals, child development is increasingly being recognized as fundamental to addressing inequities and ensuring the right of every individual to realize their true potential [12,13]. This is a moment in which we, as a community deeply concerned about the negative impact of STH on growth, cognition, and educational attainment, can help to better define the metrics needed to evaluate early childhood development and to measure and document the positive impacts of deworming on these outcomes.

The scientific debate that has resulted from the controversy surrounding the benefits of deworming is healthy and should be encouraged. We should redirect the passion and energy away from unproductive criticism and toward solutions that will ultimately serve our community best. We should not argue that evidence for benefit from deworming should be judged by different criteria than other public health interventions. This is a moment of truly unique opportunity for the STH community. Let us come together to better define, design, demonstrate, and deliver the evidence needed to document the important gains provided by deworming.
